# Medical Items

**Published:** 1916-10

**Authors:** 


### MEDICAL ITEMS

### WHY ATTENTION TO SURGICAL LUBRICATION PAYS.

From the standpoint of results, certain lubricants are much more effective and useful than others. Thus if one lubricant is more slippery than another, it means the easier and infinitely less painful passing or the sound or catheter. This not only enables the physician to accomplish his purpose more satisfactory to himself, but in a way that naturally appeals to the patient and convinces him of his physician's skill.

Again, a non-greasy, water-soluble lubricant means non-interference with the effect of subsequent urethral medication or irrigation, so that the physician is able to obtain results that are impossible if he uses a grease that obviously prevents the proper application of local remedies.

In using the cystoscope, furthermore, greasy substances are highly objectionable since they have a most annoying way of clouding the lens and blurring and distorting the field. But a transparent, non-greasy lubricant not only permits the instrument to be passed with less pain and discomfort to the patient, but without making matters more difficult for the examiner by fogging his lens. Through its use, therefore, the physician can make a thorough examination and with maximum convenience to himself and minimum discomfort and danger to his patient, obtaining the information necessary to enable him to give the exact treatment needed.

There is another phase of the subject which many physicians are apt to overlook, and that is the patients of discriminating habits dislike to have their linen stained or soiled with a greasy, unclean looking lubricant, particularly if it can be avoided—and it surely can if a lubricant is used that is non-greasy and free from substances that stain or discolor.

The foregoing may seem small, inconsequential details, but it is by attention to the little things that many earnest, painstaking medical men not only obtain better results, but in addition win the gratitude and "good will" of their patients. It is this gratitude and "good will" that lighten the physician's every day labors and reconcile him to the trials and tribulations of medical practice. Also, here is the only "ethical" way the doctor has at his command to gain patients. "Good will" PAYS. And it pays to use anything that will contribute to that good will.*

*Do you know "K-Y" Lublicating Jelly? If not, write for a sample. Van Horn and Sawtell, 15-17 East 40th Street, New York City.

---

### TYPHOID FEVER: A RATIONAL TREATMENT.

"In the treatment of typhoid fever, what is necessary?" asks a medical writer, who proceeds to answer his own question in this wise:

"1. Endeavor to cut short the course of the attack and to lessen the danger-period during which there is risk of complications.

"2. Meet any complication which may arise, and be ready with the indicated treatment in the event of such complications.

"3. Guard against the danger of relapse by prolonging treatment beyond the period of symptoms and by general supervision during convalescence.

"4. Demand rest in bed and a milk diet, with unsweetened lemonade or barley water.

"5. Combat the effects of the toxemia from the infecting organisms by administering Typhoid Phylacogen."

Typhoid fever, as is well known, is an acute infectious disease, due to the entrance into the body of the bacillus of Eberth, commonly designated the bacillus typhosus. And while this bacillus is recognized as the specific cause, it is conceded that complicating organisms, as the bacillus coli communis, the bacillus dysenteriae, the paracolon bacillus, the pneumococcus, the staphylococci and the streptococci, may play an appreciable part in the disease process.

In view of these facts, treatment with Typhoid Phylacogen would seem a rational procedure, this phylacogen consisting of a culture filtrate of the bacillus typhosus of Eberth and Mixed Infection Phylacogen. In support of the treatment it is said that a marked effect in all favorable cases is the comparatively prompt subsidence of the fever and the early establishment of convalescence. It is also pointed out that, while shortening the disease-period, this therapy also simplifies treatment. It consists ordinarily of one injection a day and does away with ice, the bath tub and supplementary attendants. For the technique of administration, suggestions as to dosage, etc., physicians are referred to the pamphlet "Typhoid Phylacogen," issued by Parke, Davis & Co., a comprehensive booklet containing information that cannot fail to be of interest and value to any practitioner.

---

### COD LIVER OIL IN HOT WEATHER.

In Cord. Ext. Ol. Morrhuae Comp. (Hagee) the clinician who seeks the utmost from his therapeutic and dietetic aids, will find the ideal cod liver oil preparation for hot weathe. Patients who rebel against the less palatable emulsions and complain of continued intolerableness, will take Cord. Ext. Ol. Morrhuae Comp. (Hagee) over long periods without being subjected to the slightest disagreeable gastric symptoms. In securing this extraordinary degree of palatability for their product the manufacturers of Cord. Ext. Ol. Morrhuae Comp. (Hagee) have not sacrificed the slightest therapeutic or nutritive effect of the oil, for their process of manufacture, whilst eliminating the grease, retains in the finished product the essential elements of the oil.

---

### NERVOUS IRRITABILITY OF CHILDREN.

During the summer season the physician often must have recourse to sedatives in the case of his little patients, who may be suffering from the ailments of the season. When this becomes necessary he will find PASADYNE (Daniel) to be a highly suitable agent for the purpose, for not only is it therapeutically active but also it is innocuous. It is this latter feature that gives PASADYNE (Daniel) a position superior to that of the other sedatives. PASADYNE (Daniel) is simply a distinctive name for a pure concentrated tincture of passiflora incarnata, made for a generation by one firm specializing in its manufacture. A sample bottle may be had by addressing the laboratory of John B. Daniel, Inc., Atlanta, Georgia.

---

The ampoule has attained a prominent position as an aid to medication, especially where subcutaneous, intramuscular and intravenous injections are required for immediate and prompt relief; the ampoule has also made it possible to administer with celerity accurate accounts of sterile solutions of definite strength and known therapeutic value.

It is all important that solutions for hypodermatic injections should be sterile; it is likewise evident that physicians are very frequently unable to prepare sterile solutions extemporaneously. To date no container has proven as practical as the ampoule.

Lilly Ampoules are encased in strong paper tubes, and may be conveniently carried in the pocket or medicine case. Each individual ampoule and each tube is fully labeled, making it unnecessary to keep the original container for identification.

The Lilly line of ampoules is said to meet the requirements of the most exacting clinican both in quality and range of items listed.

---

The Georgia Society of the City of New York desires information regarding the names and addresses of all Georgians at present residing in New York City. If the readers of this paper will forward such information as they have to Powell Crichton, Secretary of Georgia Society, at No. 120 Broadway, New York City, the information will be used for the purpose of making a complete list as well as to add to the membership of the Society.

### CO-OPERATIVE COMMUNITIES AMONG NATIVE ALASKANS.

Under the guidance of the teachers in the Government schools of Alaska, co-operative community enterprises have been sucessfully established in the past three or four years. During his administration President Wilson has reserved, by executive order, a number of carefully selected tracts of land on which the natives of Alaska can obtain fish and game and conduct their own industrial and commercial enterprises without the interference of the white man.

Hydaburg, in southeastern Alaska, is a typical example of what enlightened Government policy under Secretary Franklin K. Lane has done for Alaska. This town was formed by the migration of natives from two villages to a site reserved by executive order. Here, under the supervision of the teacher of the United States public schools, a co-operative company of the natives was organized to transact the mercantile business of the settlement and to operate a sawmill, the machinery for which was provided for by the Government.

The people of Hydaburg, through their own efforts, have turned a dense forest into a thriving town with a busy wharf, a sawmill that turns out good lumber for them at a cost of $10.00 per thousand, neat, single-family homes instead of the communal houses of the old villages, a long-boarded street of which they are proud as the finest in Alaska, and a co-operative store which has paid handsome dividends from the start.

### WHEN THE PHYSIOLOGIC PROCESSES OF THE BOWEL NEED STIMULATING.

In this day of extremes, the practitioner must not let the success obtained in certain cases of bowel stagnation, by the use of "intestinal lubrication" blind him to the fact that paraffin oil is essentially restricted in its indications. To employ it indiscriminately in all cases of constipation means complete failure to get results in many instances—and the consequent discrediting of a remedy of undoubted value when properly used.

As a matter of fact, in a large proportion of cases of constipation there is atonicity of the muscular coat of the intestines, together with marked decrease of glandular activity. Measures to impart tone to the bowel musculation and increase the glandular secretions are therefore imperative and no remedy has been found more ffective for these two main purposes than Prunoids. This has provn itself a true corrective of constipation of functional origin, its effect on the physiologic processes of the bowels not only assuring a prompt restoration of intestinal activity, but with gratifying freedom from all griping or reactionary constipation. The most casual test will show Prunoids to be a true physiologic laxative that can be used with every confidence in the permanency of its benefits.

### SLEEP.

The physician has at his command numerous remedies with which sleep can be produced; some compelling—some gently persuasive, some habit-forming—some non-habituating; some dangerous—others safe. It is an eloquent commentary upon its value as a routine hypnotic that many physicians invariably select Neurosine from this wide field as the remedy to be employed. Their reasons are found: First, in the fact that with Neurosine they can depend on producing sleep, sleep closely approximating the natural. Second, no depression or other unpleasant sequelae attends the awakening. Third, it is safe. There is no fear of habit-forming or immediate evil effect.

Combining, as it does, maximum potency with perfect safety, Neurosine is the logical and most satisfactory hypnotic for routine use. The formula and trial quantity sufficient to demonstrate these facts will be mailed free to any interested physician by the Dios Chemical Co., St. Louis, Mo.

### THE HYPERSUSCEPTIBILITY OF CHILDREN TO OPIUM.

The hypersusceptibility of children to opium is one of the most potent reasons for employing a substitute in its place in the treatment if diseases of children. It has been found that PAPINE (Battle) is well borne by children to whom opium or morphine was intolerable, but when it is remembered that in the manufacture of PAPINE, through a special process, the narcotic and convulsive elements of opium have been eliminated, the reason for this point of PAPINE'S superiority over opium, will be well understood.

PAPINE (Battle), as is well known, is a product of opium subjected to a process which while retaining the analgesic and sedative properties of the drug, separates from it its objectionable qualities, leaving the finished product of more than ordinary worth as an opiate for use in children.

### SLUGGISH, OVERLOADED BOWELS.

When the bowels are sluggish and overloaded, the whole system is usually depressed and deranged by the retention of toxic waste material. Without delay it becomes necessary to increase the activity of the bowels and promote regular evacuation of their contents. For these purposes there is no remedy that will give more prompt and satisfactory results—with such freedom from griping or after-effects as Prunoids. One to three at bedtime will afford prompt relief—without the usual cathartic discomfort—and rapidly restore functional regularity of the bowels. As one prominent physician has said, "I use Prunoids because it **regulates** as well as **evacuates** the bowels." Samples will be sent on request to the Sultan Drug Co., St. Louis, Mo.

### CHRONIC CARDIAC DISEASE.

The patient with organic heart disease needs constant attention to his digestion and nutrition. A little falling off in the general vitality and the compensatory status of the heart may be lost forever. At the first sign of nutritional decline, therefore, Gray's Glycerine Tonic Comp. should be given. This dependable tonic braces a wavering organism by reinforcing weakened functions. Thus it not only directly contributes to cardiac power, but what is vastly more important, materially reduces the stress under which a feeble heart constantly has to labor. In other words, while increasing the carrying power of the heart, it also decreases the burden.

---

### PERSISTENT COUGHS AND COLDS.

Colds that linger invariably owe their persistence to inability of the body to exert sufficient resistance to overcome germ activity. Recovery, in consequence, is always largely a question of raising the general vitality and increasing bodily resisting power.. To accomplish this, no remedy at the command of the profession is so promptly effective as Gray's Glycerine Tonic Comp. Under the use of this dependable restorative and reconstructive, the appetite is increased, the digestion improved, the nutritional balance restored, and the vital resistance so raised that the body can control infectious processes, and establish a safe and satisfactory convalescence.

In the treatment of colds, therefore, "Gray's" can be relied upon to raise the defensive forces of the organism and fortify it against germ attack.

---

### REPRINTS

Of Original Articles published in the Journal-Record of Medicine will be furnished at the following prices:

**500 Copies 50c per page.**

**1000 Copies 75c per page.**

When Reprints are desired it should be indicated on the copy.

### SAL HEPATICA

**AN EFFERVESCING**

**SALINE COMBINATION**

**LAXATIVE AND ELIMINANT**

BRISTOL-MYERS CO.
NEW YORK

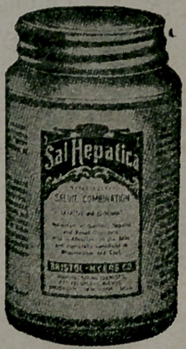

### CONTROLLING THE NERVOUS ELEMENT IN FEMALE DISEASES.

Utero-ovarian congestion, traceable to extreme irritability of the local nervous mechanism, invariably requires appropriate antispasmodic treatment. Obviously great care, however, must be exercised in selecting the measures to be employed. Among the sedatives that have been found serviceable, none has proven more effective or given more uniform satisfaction in every particular than Peacock's Bromides. Many clinicians have learned to appreciate the antispasmodic properties of this preparation, and as a consequence Peacock's Bromides have long filled an important place in gynecologic therapeutics. Efficient, reliable and remarkably free from any unpleasant effects, this dependable combination of carefully selected bromide salts is of exceptional utility in female disorders in which the nervous element is prominent. In these conditions, it can be used with every confidence, not alone in its therapeutic efficiency, but what is often quite as important, in its notable freedom from gastric disturbance or other unpleasant effect. A particularly gratifying feature of Peacock's Bromides is its capacity to relieve pain and discomfort without inducing a drug habit, a result which the opiates and so many other pain relieving remedies all too often produce. Peacock's Bromides surely fill an important place in the therapy of the painful ills of womankind.

---

### THE HEART IN SUMMER.

The heart and circulatory dangers common to hot weather, particularly in the aged and infirm, can be easily avoided by the systematic use of Gray's Glycerine Tonic Comp.

Its administration in two to four teaspoonful doses throughout the hot season ads digestion, tones the nervous system, strengthens the heart, and goes far towards maintaining a safe circulatory balance. A very safe and refreshing way to give "Gray's" is in iced water, or with cracked ice.

